# Modeling Temperature-Dependent Development of *Glyphodes pyloalis* (Lepidoptera: Pyralidae)

**DOI:** 10.1093/jisesa/iex001

**Published:** 2017-02-28

**Authors:** Zohreh Moallem, Azadeh Karimi-Malati, Ahad Sahragard, Arash Zibaee

**Affiliations:** Department of Plant Protection, Faculty of Agricultural Sciences, University of Guilan, Rasht, Iran

**Keywords:** degree day, *Glyphodes pyloalis*, temperature threshold, thermal model

## Abstract

Development of *Glyphodes pyloalis* Walker was studied under laboratory conditions at constant temperatures of 12, 16, 20, 24, 28, 30, 32, and 36 °C. No development occurred at 36 °C. Although eggs hatched at 12 ºC, no larvae were capable of developing to adult stage. At 16 ºC, survival rate was low (4%) and prepupal stage lasted 101.68 ± 11.03 d. Larvae completed development through six stadia at 16, 30, and 32 °C. Developmental time of overall immature stages varied from 46.62 d at 20 °C to 22.04 d at 30 °C and increased at 32 °C. The lower temperature thresholds of 10.30 and 11.22 °C, and thermal constants of 429.18 and 401.88 DD were estimated by traditional and Ikemoto–Takai linear models, respectively. The *T*_min_ values estimated by Analytis, Briere-2, Lactin-2, and Sharpe–Schoolfield–Ikemoto (SSI) for overall immature stages were 12.40, 12.92, 9.00, and 13.04 °C, respectively. The fastest development temperatures (*T*_fast_) of 31.1, 31.1, 30.8, and 30.7 °C were estimated for overall immature stages based on Analytis, Briere-2, Lactin-2, and SSI, respectively. The intrinsic optimum temperature (*T*_opt_) estimated from the thermodynamic SSI model for total developmental time was 24.63 °C, in which the maximal active state enzymes involved in developmental process. The nonlinear models of Analytis, Lactin-2, Briere-2, and SSI estimated the upper temperature thresholds (*T*_max_) at 36.66, 35.97, 38.88, and 34.05 °C, respectively. These ﬁndings could be used to predict the population dynamics of *G. pyloalis* for an effective management.

The lesser mulberry pyralid, *Glyphodes pyloalis* Walker (Lepidoptera: Pyralidae), is a specialist pest on mulberry (*Morus* spp.) and is widely distributed throughout Asia, where the species causes serious damage to sericulture not only by its larval grazing on leaves but also by transmission of some viral diseases infectious to the silkworm (Watanabe et al. 1988, Madyarov et al. 2006). On the other hand, *G. pyloalis* becomes a major pest of mulberry as shade trees in urban area (Kumar et al. 2002). Recently, this pest has caused severe damage to mulberry plantations in northern Iran especially Guilan province. The leaf area eaten by the first and second instar larvae is negligible, but feeding increases in later instars, and fifth instar larvae feed whole leaf and finally only the ribs remain (Khosravi and Jalali Sendi 2010). Since the larvae of the pest defoliate mulberry and finally lead to plant death, some investigations were done to know its biological parameters and control tools. Khosravi and Jalali Sendi (2010) studied the demographic parameters of *G. pyloalis* and its behavioral aspects. Yazdani et al. (2014) stated that the essential oils of *Thymus vulgaris* L. and *Origanum vulgare* L. were effective for *G. pyloalis* control through disturbance on activity of macromolecules, digestive and detoxifying enzymes. Moreover, the effects of different mulberry varieties on the nutritional indices of *G. pyloalis* larvae (Oftadeh et al. 2014), as well as its life table parameters (Oftadeh et al. 2015) were determined under laboratory conditions. Furthermore, the influence of abiotic climate factors on incidence and severity (Ramegowda et al. 2012) and damage rate of *G. pyloalis* (Borgohain et al. 2015) was described to evolve a successful IPM program. According to Borgohain et al. (2015), the evening relative humidity and minimum temperature had significant positive effects on occurrence of *G. pyloalis.*

It should be considered that among the climatic factors, temperature is the most important, as it has profound influence on the development and survival of insects. The insect developmental rate, as poikilothermic organism, is affected by the temperature to which insects are exposed (Davidson 1944, Campbell et al. 1974). In fact, temperature is a critical factor that influences pest biology, distribution and abundance, as well as its population dynamics (Braman et al. 1984, Tobin et al. 2003, Zahiri et al. 2010). Attempts to quantify the effects of temperature on developmental rate, growth, fecundity and enzyme activities have been carried out by several studies for different insect and mite species (Kontodimas et al. 2004, Zahiri et al. 2010, Jafari et al. 2012, Karimi-Malati et al. 2014). In addition, a variety of temperature-driven rate models have been proposed to describe the relationship between temperature and insect development (Sharpe and DeMichele 1977; Analytis 1981; Schoolfield et al. 1981; Lactin et al. 1995; Briere et al. 1999; Shi et al. 2011a,b). Several studies revealed that there is no development at temperatures below the lower threshold. While as temperature rises, developmental rates increase up to an optimum temperature, above which they again decrease and eventually cease at their upper threshold (Sharpe and DeMichele 1977, Analytis 1981, Briere et al. 1999). Based on linear models, lower temperature threshold and thermal constant can be estimated at moderate temperatures. However, linear models proved unsecure in predicting developmental rate near extreme conditions, therefore, nonlinear models have been proposed to describe developmental rate response curves over the broad range of temperatures (Wagner et al. 1984).

Although different climate factors affecting occurrence and infestation of *G. pyloalis* were considered (Ramegowda et al. 2012, Borgohain et al. 2015), no information exists on the relationship of its developmental rate and temperature. This study was conducted to assess the developmental rate of *G. pyloalis* at eight constant temperatures and estimate the temperature thresholds and thermal requirements, which would be useful in developing models for predicting its distribution and abundance. Predicting the seasonal occurrence of *G. pyloalis* based on climate factors such as temperature is essential for its accurate scheduling of census samples and control tactics. Two linear and four nonlinear models were used for estimating accurate thermal constant and temperature thresholds of *G. pyloalis*, which would be useful in developing phenological models and constructing an effective pest management program.

## Materials and Methods

### 

#### Rearing Methods

The larvae of *G. pyloalis* were collected from mulberry trees in Rasht, Guilan province, Iran during 2015. They were reared at laboratory conditions at 25 ± 1 °C and 70 ± 10% RH, with a photoperiod of 16:8 L:D h on fresh mulberry leaves. To obtain the same aged eggs, female and male moths (15 pairs) were kept inside the oviposition containers (50 × 50 × 50 cm) with a 10% honey solution on cotton wool for feeding and mulberry leaves for oviposition.

#### Development and Survivorship of Immature Stages

After mating and oviposition, one hundred to 300 (depending thermal treatment) freshly laid eggs (< 24 h old) were transferred to plastic boxes (18 × 15 × 7 cm) with wet cotton wool in growth chambers at eight constant temperatures of 12, 16, 20, 24, 28, 30, 32, and 36 ± 1 °C at 70 ± 10% R.H. and photoperiod of 16:8 L:D h. Changing in shape and color of eggs was monitored daily under the stereomicroscope. Incubation period and hatching rate were recorded.

The newly hatched larvae were placed individually in plastic containers (7 × 8 × 3 cm) with a hole in their lids covered by a ﬁne mesh to provide ventilation. The petioles of mulberry leaves were kept in tubes containing water to keep the cutting leaves as fresh as possible. The leaves were replaced every other day for larvae at 16–24 °C and daily for those larvae at 28–32 °C because plant desiccation occurred faster than lower temperatures. The larvae were checked and the instars were regularly recorded using the exuviae of larval head capsules. The matured larvae changed color from green to purple and began making fine cocoons considering prepupal stage. After pupation, they were slipcovered and sexes were determined based on morphological characters of pupal last abdominal segment. After that they were replaced in their fine cocoons. The cocooned pupae were checked and the emerged adults recorded daily. Developmental time of different and overall immature stages was recorded based on regular observations with 24 h intervals.

#### Thermal Models

The reciprocal of developmental time for different stages of *G. pyloalis* was calculated to obtain the developmental rate. Two linear, traditional and Ikemoto–Takai models were applied to estimate the temperature-dependent development of egg, larval, prepupal, pupal, and total immature stages of *G. pyloalis* on mulberry leaves. The traditional and Ikemoto–Takai models are as follows, respectively:

(1) $$\frac{1\;}{D}=-\frac{T_{min}}{K}+\frac{T}{K}$$

(2) $$DT=K+T_{min}D$$

where *D* is the duration of development (days), *T* is the ambient temperature, *T*_min_ is the lower temperature threshold, and *K* is the thermal constant (degree day, DD).

The latter function is a new linearized formula was proposed by Ikemoto and Takai (2000). Ikemoto and Takai (2000) particularized some problems regarding the traditional linear model would result in a lower *T*_min_ and larger *K*, hence, equation (2) is derived from the traditional linear model to obtain more reliable estimates of the parameters.

It should be considered that the relationship between temperatures and developmental rate is curvilinear near lower and upper temperature thresholds. To describe the developmental rate over a wider temperature range, four nonlinear models including Analytis, Briere-2, Lactin-2, and Sharpe–Schoolfield–Ikemoto (SSI) were chosen (Analytis 1981, Lactin et al. 1995, Briere et al. 1999, Shi et al. 2011b). These four mentioned nonlinear formulations are as follows, respectively:

(3) $$\frac{1}{D}=a\times {\left(T-T_{min}\right)}^{n}\times {\left(T_{max}-T\right)}^{m}$$

(4) $$\frac{1}{D}=a\times T(T-T_{min})\times {\left(T_{max}-T\right)}^{1/d}$$

(5) $$\frac{1}{D}=\exp\left(p\times T\right)-\exp\left(p\times T_{max}-\left(\frac{T_{max}-T}{\Delta T}\right)\right)+\lambda$$

where *T*_min_ is the lower temperature threshold, *T*_max_ is the upper temperature threshold, *a*, *d*, *n*, *m, p, λ*, and *ΔT* are ﬁtted coefﬁcients (Analytis 1981, Briere et al. 1999, Roy et al. 2002, Kontodimas et al. 2004). In addition, the SSI model was used in this research which is closely related to the impact of temperature on the enzyme. Using SSI model enable researchers to estimate the intrinsic optimum temperature (*T*_opt_) in which the population size is maximal with a low mortality (Ikemoto 2005, 2008, Shi et al. 2011b). Ikemoto (2005) and Shi et al. (2011b) demonstrated that the intrinsic optimum temperature (*T*_opt_) should represent a temperature at which the mortality of insects is very low, and that the net reproductive rate is generally highest. In fact, *T*_opt_ is different from *T*_fast_ that make insects develop fastest within shortest duration:

[6] $$\frac{1}{D}=\frac{\rho_{\Phi \;}(T/T_{opt})\;\times exp[{\Delta H}_{A\;}/R\times ((1/T_{opt})-(1/T))]\;}{1+exp[{\Delta H}_{L\;}/R\times ((1/T_{L})-(1/T))]+exp[{\Delta H}_{H}/R\times ((1/T_{H})-(1/T))]}$$

where *ρ_Φ_* is the mean developmental rate at *T*_opt_ (1/*d*), *T*_opt_ is the intrinsic optimum temperature at which the probability of an enzyme being in the active state is maximal. Δ*H_A_*, Δ*H_L_*, and Δ*H_H_* are the enthalpy of activation of the reaction that is catalyzed by the enzyme (cal/mol), the change in enthalpy associated with low temperature inactivation of the enzyme (cal/mol), and the change in enthalpy associated with high temperature inactivation of the enzyme (cal/mol), respectively, *R* is the gas constant (1.987 cal/deg/mol), *T_L_* is the temperature at which the enzyme is 1/2 active and 1/2 low temperature inactive, and *T_H_* is the temperature at which the enzyme is 1/2 active and 1/2 high temperature inactive (Both in Kelvin degrees).

Since running the SSI model through Ikemoto (2008) takes 3 h for an average personal computer, a modiﬁed mentioned program was proposed by Shi et al. (2011b) to speed up the estimation of model parameters.

#### Critical Temperatures and Parameter Estimation

Critical temperatures and thermal requirement of *G*. *pyloalis* were estimated by above-mentioned models.

The lower temperature threshold (*T*_min_), the temperature below which different stages did not develop. The standard error (SE) of *T*_min_ calculated from the linear models is

[7] $${SE}_{T_{min}}=\frac{r}{b}\times \sqrt{\frac{S^{2}}{{N\times r}^{2}}+{\left(\frac{{SE}_{b}}{b}\right)}^{2}}$$

where *S*^2^ is the residual mean square of *r*, *r* is the sample mean, and *N* is the sample size (Campbell et al. 1974, Kontodimas et al. 2004).

The upper temperature threshold (*T*_max_), the temperature above which the life cannot be maintained for any significant period (Kontodimas et al. 2004). This value was estimated only by the nonlinear models.

The fastest development temperature (*T*_fast_), defined as the temperature at which the highest developmental rate was recorded. However, the ﬁtness of population is usually not maximal because of the higher mortality at *T*_fast_.

Thermal constant (*K*), the amount of thermal energy (DD) needed to complete development of different stages. The thermal constant can be estimated only by the linear equation. The SE of *K* was estimated by using the following equation (Campbell et al. 1974, Kontodimas et al. 2004).

[8] $${SE}_{K}=\frac{{SE}_{b}}{b^{2}}$$

#### Statistical Analysis

Normality of distribution was checked with the Kolmogorov–Smirnov test before comparative analyses were performed. Effect of temperature on developmental periods of *G. pyloalis* was analyzed by one-way analysis of variance ANOVA (PROC GLM, SAS Institute 2007) and means were separated using Tukey Honestly Significant Difference HSD multiple comparison (*P* ≤ 0.01). The linear models were analyzed using statistical software MINITAB 16.0 and nonlinear models analyzed using linear and nonlinear platforms of JMP, v 7.0 (SAS Institute 2007). For estimating the parameters of the SSI model, a program which runs on R software was used at the present study (Shi et al. 2011b).

## Results

### 

#### Developmental Time and Mortality

The mean developmental time of each immature stage of *G. pyloalis* at six constant temperatures are shown in Table 1. The results of developmental time and survival rate showed that *G. pyloalis* was able to complete its life cycle and development at a wide range temperature. In fact, the adults were capable of emergence across a range of 20–32 °C, whereas few eggs developed to adult stage at 16 ºC (with 4% survivorship; only six emerged adults). As far as prepupal stage is concerned, at 16 ºC *G. pyloalis* required 101.68 ± 11.03 d to develop to pupal stage maybe due to stop developing (or diapause occurrence) in prepupal stage. For these two reasons, too long duration and low survivorship of prepupal stage both occurred at 16 °C, developmental times of prepupal, pupal, and total immature stages were ignored and comparing the mean duration of above-mentioned stages (prepupal, pupal, and total developmental times) was done without considering of the temperature at 16 °C (Table 1).

**Table 1.** Developmental time (means ± SE) and survival of *Glyphodes pyloalis* immature stages at constant temperatures

	Temperature (°C)					
Stage	16	20	24	28	30	32
Egg	9.75 ± 0.07a	6.52 ± 0.04b	4.76 ± 0.04c	3.71 ± 0.05d	3.00 ± 0.00e	3.00 ± 0.00e
no (s)	150 (65.71)	112 (70.38)	107 (83.53)	80 (87.50)	106 (77.36)	84 (61.90)
Larva I	8.92 ± 0.18a	4.97 ± 0.09b	3.15 ± 0.07c	2.38 ± 0.10d	2.00 ± 0.00e	2.45 ± 0.10d
no (s)	97 (91.75)	78 (85.90)	89 (100)	59 (100)	82 (100)	52 (100)
Larva II	6.48 ± 0.14a	3.68 ± 0.10b	2.41 ± 0.06c	2.00 ± 0.04d	1.04 ± 0.03e	1.84 ± 0.10d
no (s)	89 (94.38)	67 (98.51)	89 (97.75)	59 (94.92)	82 (100)	52 (100)
Larva III	6.18 ± 0.13a	4.00 ± 0.12b	2.15 ± 0.05c	2.06 ± 0.03cd	1.76 ± 0.06d	1.95 ± 0.10cd
no (s)	84 (90.48)	66 (93.94)	87 (97.70)	56 (100)	82 (95.12)	52 (98.08)
Larva IV	6.72 ± 0.17a	5.53 ± 0.15b	2.64 ± 0.07c	2.44 ± 0.07cd	1.88 ± 0.05e	2.00 ± 0.08de
no (s)	76 (85.53)	62 (96.77)	85 (98.82)	56 (94.64)	78 (94.87)	51 (100)
Larva V	8.22 ± 0.16a	6.18 ± 0.13b	3.72 ± 0.08c	2.87 ± 0.09d	3. 07 ± 0.07d	2.74 ± 0.16d
no (s)	65 (83.08)	60 (100)	84 (96.43)	53 (98.11)	74 (93.24)	51 (86.27)
Larva VI^a^	8.33 ± 1.33a	–	–	–	4.50 ± 1.15b	2.67 ± 0.19b
no (s)	54 (92.59)	–	–	–	69 (98.55)	44 (86.36)
Larvae	37.02 ± 0.34a	24.37 ± 0.31b	14.06 ± 0.12c	11.75 ± 0.13d	10.16 ±0.18e	11.89 ± 0.28d
no (s)	97 (51.55)	78 (76.92)	89 (91.01)	59 (88.14)	82 (82.93)	52 (73.08)
Pre pupa^b^	101.68 ± 11.03	4.64 ± 0.11a	2.28 ± 0.05b	1.94 ± 0.03c	2.11 ± 0.06bc	2.25 ± 0.19bc
no (s)	50 (38)	60 (83.33)	81 (96.30)	52 (100)	68 (92.65)	38 (84.21)
Pupa^b^	24.67 ± 1.61	12.32 ± 0.14a	8.62 ± 0.07b	7.08 ± 0.05c	6.80 ± 0.09c	6.14 ± 0.08d
no (s)	19 (31.57)	50 (94)	78 (87.18)	52 (98.08)	63 (85.71)	32 (65.63)
Immature^b^	134.33 ± 25.19	46.62 ± 0.23a	29.34 ± 0.17b	24.47 ± 0.17c	22.04 ± 0.20d	22.43 ± 0.25d

No, sample size; s, survival (%). Means within rows followed by the same letters are not signiﬁcantly different (*P* < 0.05).

a At 20, 24, and 28 °C larvae completed development in five stadia.

b Comparing the prepupal, pupal, and total developmental times was done without considering of the temperature at 16 °C.

According to our results, eggs could hatch after 17.72 ± 0.86 d at 12 °C without any surviving to the next stage and all neonate larvae died. In addition, at 36 °C, no eggs hatched. Developmental time for each stage was significantly influenced by temperature: incubation period (*F* = 2961.97; df = 5, 742; *P* < 0.0001), larval (*F* = 1941.75; df = 5, 348; *P* < 0.0001), prepupal (*F* = 173.14; df = 4, 274; *P* < 0.0001), pupal (*F* = 670.42; df = 4, 240; *P* < 0.0001), and overall immature stages (*F* = 2479.46; df = 4, 240; *P* < 0.0001) (Table 1). The larval developmental time ranged 24.37 ± 0.31 to 10.16 ± 0.18 d at 20 and 30 ºC, respectively. Moreover, comparing the number of stadia in larval stage indicated that an extra (sixth) stadium was observed at extreme temperatures (16, 30, and 32 °C). In fact, no larvae required more than ﬁve stadia at 20, 24, and 28 °C.

The survival rate of overall immature stages indicated that the lowest survival rate occurred at 16 °C (4%). Although egg hatching occurred at 12 °C, all neonate larvae died due to the exposure to low temperature. Furthermore, survival of total larval stage of *G. pyloalis* at six constant temperatures revealed that the survival was highest (65%) at 28 °C, followed by 24 °C (Table 1).

#### Model Evaluations

**Fig. 1.**
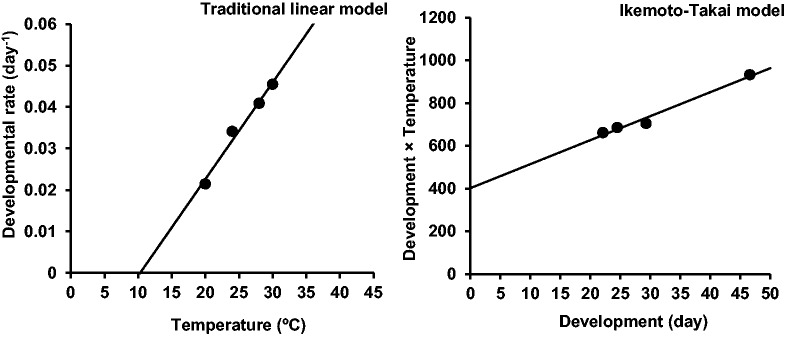
Fitting the linear models (line) to observed developmental rates (•) of *Glyphodes pyloalis*.

The developmental rate of *G. pyloalis* increased linearly within the examined temperature range (20–30 °C). Developmental time at >30 °C (32 °C) was outside the linear segment of the growth curve and therefore excluded from the linear regression. Results of parameter estimation of linear models (traditional and Ikemoto–Takai), coefficients of determination (*R*^2^ and *R*^2^_adj_), lower temperature thresholds and thermal constants are presented in Table 2. The estimated lower temperature thresholds for total developmental time were 10.30 and 11.22 °C, while the thermal constants were 429.18 and 401.88 DD, using the traditional and Ikemoto–Takai linear models, respectively. The thermal requirements were lowest at the prepupal stage and the highest at the larval stage. The curves of influence of temperature on developmental rate of overall immature stages fitted by two linear models are shown in Fig. 1.

**Table 2.** Lower temperature threshold (*T*_min_ ± SE) and thermal constant (*K ± *SE) of immature stages of *Glyphodes pyloalis* estimated by linear models

Stage	Regression equation	*R*^2^%	*R*^2^_adj_%	*P*	*T*_min_	*K*
Traditional linear						
Egg	1/*D* = −0.197 + 0.0172 *T*	97.10	95.70	0.014	11.45 ± 1.77	58.14 ± 7.07
Larvae	1/*D* = −0.0658 + 0.00548 *T*	97.60	96.40	0.01	12.01 ± 1.54	182.48 ± 20.16
Prepupa	1/*D* = −0.508 + 0.0374 *T*	92.65	85.30	0.11	13.58 ± 5.87	26.74 ± 13.87
Pupa	1/*D* = −0.0786 + 0.00801 *T*	99.14	98.70	0.004	9.81 ± 2.02	124.84 ± 16.27
Immature	1/*D* = −0.0240 + 0.00233 *T*	98.06	97.09	0.009	10.30 ± 1.58	429.18 ± 43.09
Ikemoto–Takai linear						
Egg	*DT* = 60.278 + 10.977 *D*	97.23	95.85	0.013	10.98 ± 1.3	60.28 ± 6.14
Larvae	*DT* = 170.22 + 12.89 *D*	98.68	98.02	0.006	12.89 ± 1.05	170.22 ± 16.92
Prepupa	*DT* = 23.19 + 14.9 *D*	98.86	97.71	0.06	14.93 ± 1.60	23.19 ± 5.12
Pupa	*DT* = 124.14 + 9.89 *D*	98.81	98.21	0.006	9.89 ± 0.76	124.14 ± 6.79
Immature	*DT* = 401.88 + 11.22 *D*	97.93	96.89	0.01	11.22 ± 1.15	401.88 ± 37.04

Developmental times at 16 and 34 °C were excluded from linear regressions.

**Fig. 2.**
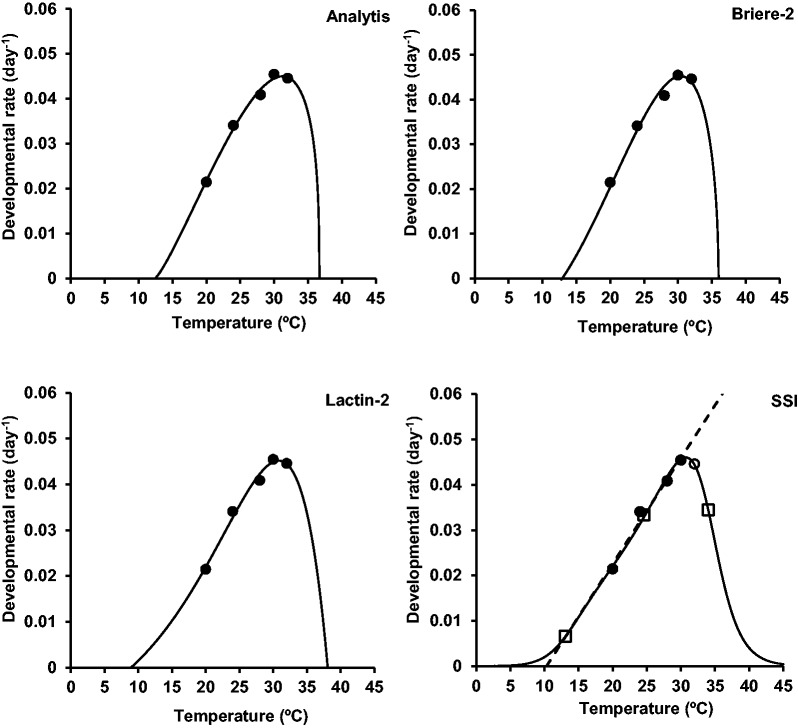
Fitting the nonlinear models to observed developmental rates of *Glyphodes pyloalis*. (•) observed data. In SSI model (○) indicates data points outside the range of the linear model. (□) denotes the predicted developmental rates at *T_L_*, *T*_opt_, and *T_H_*.

Four nonlinear models (Analytis, Briere-2, Lactin-2, and SSI) were fitted to the data on developmental rate of egg, larval, prepupal, pupal, and overall immature stages of *G. pyloalis* at the temperature range from 20 to 32 °C (Table 3). The values of *R*^2^_adj_ were used to determine the goodness of fit the models. The adjusted coefficients of determination (*R*^2^_adj_) in all tested models for overall immature stages were higher than 0.95. The curves of the relationship between temperature and developmental rate of total immature stages fitted by mentioned models are depicted in Fig. 2.

**Table 3.** Estimated parameters and goodness of ﬁt of the nonlinear models ﬁtting to developmental rates of *Glyphodes pyloalis*

Model	Parameters	Egg	Larva	Prepupa	Pupa	Immature
Analytis	*a*	0.0180805941	0.0093445459	0.0026737994	0.0099970236	0.00006334287
	*T*_min_	12.087979091	14.55	15.977576718	10.953642573	12.4
	*T*_max_	32	32.002136251	37.028156352	32	36.659062923
	*n*	0.9971826195	0.8459044687	1.2959553193	0.944708698	1.2456828095
	*m*	0.0020776935	0.0360820395	0.9232286618	0.0026302548	0.3586178111
	*T*_fast_	31.9	31.9	28.3	31.9	31.1
	*R*^2^_adj_	0.9711	0.9782	0.9465	0.9907	0.9898
Briere-2	*a*	0.0006457008	0.0000707783	0.0002463701	0.0002935356	0.000035978
	*d*	−4432866.14	1.7128474553	1.1378065837	−38099.29866	1.9929415702
	*T*_min_	12.85	14.159022666	16.037315313	9.575530105	12.923886455
	*T*_max_	32.111	35.649026102	36.031026187	30.77	35.968777953
	*T*_fast_	32.9	30.4	27.7	31.9	31.1
	*R*^2^_adj_	0.9529	0.9548	0.9655	0.9829	0.9817
Lactin-2	*p*	0.0125922236	0.0049437231	0.1096400671	0.0067671533	0.1238102145
	Δ*T*	0.0813549794	0.7008941528	8.687924051	0.0778880862	8.0661967712
	*λ*	−1.138739779	−1.060232293	−0.590717205	−1.063005565	−0.014783871
	*T*_max_	32.336013185	34.649397346	37.653561342	32.339177483	38.88
	*T*_min_	10.3	11.9	14.8	9.1	9.0
	*T*_fast_	31.8	30.6	28.6	31.3	30.8
	*R*^2^_adj_	0.9764	0.9682	0.9326	0.9912	0.9853
SSI	*ρ_Φ_*	0.2866211	0.06756835	0.3332183	0.1108385	0.03337244
	*T*_opt_	28.2088	24.3899	22.663	23.654	24.6338
	*T_L_*	11.13197	12.97607	16.9636	9.950553	13.0422
	*T_H_*	32.451	32.8627	31.4021	35.0202	34.0533
	Δ*H_A_*	13,292.41	15,236.67	20,317.41	12,144.71	13,205.04
	Δ*H_L_*	−76,643.64	−138,159.7	−127,233.6	−143,849.7	−73,234.83
	Δ*H_H_*	786,228.6	122,641.5	76,757.69	92,395.43	103,394
	*T*_fast_	31.5	30	29.1	31.3	30.7
	*X*^2^	0.0008029253	0.0008813896	0.0139181	0.000379911	0.000274224
	*R*^2^_adj_	0.9827	0.9324	0.8079	0.9779	0.9557

The results of present study showed that the lower developmental thresholds (*T*_min_) for overall immature stages estimated by the Analytis, Briere-2, and Lactin-2 were 12.4, 12.92, and 9 °C, respectively. Moreover, the SSI model estimated *T_L_* for overall immatures stage at 13.04 °C, in which the hypothetical enzyme was half active and half inactive. Based on current study, the Analytis, Briere-2, Lactin-2, and SSI models estimated *T*_fast_ for overall immature stages at 31.1, 31.1, 30.8, and 30.7 °C, respectively. In estimated *T*_fast_ developmental time is shortest but the ﬁtness of population is usually not maximal because of the higher mortality. Whereas the estimates of *T*_opt_ using SSI model for different stages varied from 22.66 to 28.21 °C which represented the optimal temperature for *G. pyloalis* population to develop with a low mortality.

## Discussion

The estimated temperature thresholds and thermal constants are potential indicators for developing a phenology model of *G. pyloalis* and predicting its population dynamics. According to the surveys, so far research study has not been done regarding to critical temperatures (*T*_min_, *T*_max_, *T*_opt_) and thermal requirements of the lesser mulberry pyralid. The biology of the pest was examined by Khosravi and Jalali Sendi (2010) under laboratory conditions, who demonstrated that the incubation period of *G. pyloalis* was 4.06 d at 24 ± 1 °C. Similarly working with *G. pyloalis* on four mulberry varieties, Oftadeh et al. (2015) stated that the egg incubation periods varied from 3.77 to 4.44 d at 24 ± 1 °C. Our results for incubation period of *G. pyloalis* at same temperature agreed with those reported by Khosravi and Jalali Sendi (2010) and Oftadeh et al. (2015). Moreover, Khosravi and Jalali Sendi (2010), and Oftadeh et al. (2015) reported that the larval stage of *G. pyloalis* required five stadia, however, an extra (sixth) stadium was observed at 16, 30, and 32 °C at present study. These differences may be due to larval body size depending on very high- or low-developmental rate at extreme temperatures. Shi et al. (2013) emphasized that both population size and body size are important in ﬁtness of ectotherms, indicating developmental times are based on the particular morphology and size of the species at different temperatures (Honěk 1996). Therefore, an extra molting might be justifiable for larval stage of *G. pyloalis* at extreme temperatures.

According to the present study, it should be considered that prepupal developmental times were 4.64, 2.28, 1.94, 2.11, and 2.25 d at 20, 24, 28, 30, and 32 °C, respectively, whereas at 16 °C prepupal developmental time prolonged 101.68 d. It seems that this longest prepupal developmental time observed at 16 °C may be related to less tolerance to low temperatures which could stop development and prolong this stage. Few researchers have focused on overwintering or diapause of *G. pyloalis* under field conditions. Mathur (1980) monitored life history of *G. pyloalis* during 1933–1934 and stated that the matured larvae of last generation made the cocoons and hibernated inside the leaves on the ground from mid-autumn. Based on Mathur (1980), no hibernation of pupae occurred and overwintering larvae pupated in next early March. The findings of present study confirmed that at 16 °C matured larvae developed to prepupae and prepupal developmental time lasted 101.68 d, suggesting that the overwintering matured larvae in Mathur (1980) research might be the same as prepupae. Since Mathur (1980) did not concentrate on the prepupal period of *G. pyloalis* as a distinct stage, this assumption seems to be presumable. No more information is currently available on overwintering of *G. pyloalis*, hence probability of hibernation or diapause occurrence in prepupal stage could be proposed cautiously.

The results of the current study indicated that the egg-to-adult developmental time was completed in 29.34 d at 24 °C. Based on Khosravi and Jalali Sendi (2010), total developmental time of *G. pyloalis* were 28.77 and 29.21 d for female and male, respectively. It seems that our findings for total developmental time were consistent with those of Khosravi and Jalali Sendi (2010) at 24 °C. Whereas Oftadeh et al. (2015) reported a higher value of total developmental time at 24 °C in their study wherein *G. pyloalis* completed the development from 35.04 to 37.64 d on different mulberry varieties.

As temperature exerts noticeable inﬂuence among the climate factors, by directly affecting insect phenology and distribution, most of the models that describe insect development are temperature driven (Wagner et al. 1984). Several models have been proposed to describe developmental rate response curves over the wide range of temperatures, in which the linear model has the advantage of being easy to calculate and is the only model enabling the estimation of the thermal constant (Kontodimas et al. 2004) but it can be measured only at moderate temperatures (Wagner et al. 1984). The current study showed that developmental time of different stages (egg, larva, prepupa, and pupa) of *G. pyloalis* decreased with increasing temperature from 16 to 30 °C, and came out from linear mode at 32 °C. Assuming that developmental rate of all immature stages is a linear function of temperature within the 20–30 °C range, whereas a nonlinear response occurred at extreme temperature 32 °C. Therefore, the data deviated from linearity at 32 °C was excluded from linear regressions. Moreover, developmental rate of *G. pyloalis* at 16 °C was omitted because very low survivorship (4%) was observed at this temperature.

According to the results of the present study, the lower developmental threshold for overall immature stages of *G. pyloalis* was estimated at 10.30 and 11.22 °C based on traditional and Ikemoto–Takai linear models, respectively. Both linear models had high values of *R*^2^ and *R*^2^_adj_, indicating a high degree of conﬁdence. It should be noted that the higher *T*_min_ values were estimated by traditional (13.58 °C), Ikemoto–Takai (14.93 °C), Analytis (15.98 °C), Briere-2 (16.04 °C), Lactin-2 (14.8 °C), and SSI (16.96 °C) models for prepupal stage compared with other stages, suggesting that the prepupal stage showed sensitivity to lower temperatures. With regard to higher *T*_min_ values for prepupal stage of *G. pyloalis* compared with other (egg, larval, and pupal) stages, the prepupal stage might be assigned as critical stage for diapause or hibernation. Generally, low temperature might enable to stop development and induce hibernation at prepupal stage of *G. pyloalis* as Mathur (1980) observed under field conditions. However, a continued study is necessary to determine different factors affecting diapause and overwintering of *G. pyloalis*, as well as physiological experiments for understanding the hormonal mechanisms responsible for.

The obtained results of the present study revealed that the thermal constants for overall immature stages were 429.18 and 401.88 DD estimated by traditional and Ikemoto–Takai linear models. Considering that *G. pyloalis* required high thermal constant for completion of entire immature stages, the late incidence of *G. pyloalis* during the post commercial season of mulberry could be justifiable. Ramegowda et al. (2012) and Borgohain et al. (2015) stated that the peak of incidence and severity of *G. pyloalis* were distinct during the late season and the pest damage was limited in spring crop of silkworm. In fact, those results could support our findings on high thermal constant of *G. pyloalis*, explaining some reasons for the pest prolonger in the late spring and summer. So far no information exists on temperature-dependent development of *G. pyloalis* and in the current study its critical temperatures and thermal requirements were estimated for the first time. Hence, further physiological and ecological studies would warrant to quantify the phenology of *G. pyloalis* based on thermal requirements.

Since the linear models is unsecure in predicting development in extreme temperatures, several nonlinear models provide critical temperatures such as lower and upper temperature thresholds, fastest development temperature and intrinsic optimum temperature (Analytis 1981, Schoolfield et al. 1981, Lactin et al. 1995, Briere et al. 1999, Ikemoto 2005, Shi et al. 2011b). To describe the developmental rate more realistically and over a wider temperature range, four nonlinear models (Analytis, Briere-2, Lactin-2, and SSI) have been applied in the current investigation. Based on our results, the adjusted coefficients of determination (*R*^2^_adj_) in all mentioned nonlinear models fitting to overall developmental rate were higher than 0.95, suggesting the high degree of confidence in estimated parameters. Nonetheless, to select the models which provide satisfactory fit to observed data, the *R*^2^_adj_ is not sufficient. It should be noticed that although the Lactin-2 gave a good fit to the observed data for total developmental times as indicated by the high values *R*^2^_adj_, the model underestimated the *T*_min_ values. Comparing the *T*_min_ estimated by Lactin-2 using observed total developmental rate under laboratory conditions indicated that the Lactin-2 did not provide a realistic estimate of this critical temperature. In fact, the Lactin-2 underestimated *T*_min_ at 9 °C, whereas failure of *G. pyloalis* development was observed at 12 °C. Furthermore, *T*_min_ of 12.09, 12.85, and 11.13 °C for egg stage estimated by the Analytis, Briere-2, and SSI models were strongly provided by experimental observations, in which, eggs of *G. pyloalis* could hatch at 12 °C but no neonate larvae survived and developed to next stages. The survivorship of larvae at higher temperatures compared with egg stage resulted in estimating the higher *T*_min_ values for larval stage.

Our findings revealed that the Analytis, Briere-2, and SSI models approximately provided satisfactory estimates of *T*_max_ (36.66, 35.97, and 34.05 °C, respectively) for total immature stages which are consistent with those of experimental observations. Whereas the Lactin-2 overestimated *T*_max_ values for overall immature stages of *G. pyloalis* at 38.88 °C.

Based on current study, *T*_fast_ for overall immature stages at which the highest developmental rates were estimated, ranged 30.7–31.1 °C using the Analytis, Briere-2, Lactin-2, and SSI models. These four models seem to provide realistic values of *T*_fast_ because the shortest developmental time of *G. pyloalis* was recorded at 30 °C under laboratory conditions. Many earlier researchers documented that the temperature, at which the developmental time is shortest, should be considered as the optimal temperature (Ranjbar-Aghdam et al. 2009, Zahiri et al. 2010), ignoring the intrinsic optimum temperature (*T*_opt_) has different concepts from *T*_fast_. In fact, the temperature at which the population size reaches its maximum is not the temperature (*T*_fast_) that can make insects develop fastest with low survival and net reproductive rate. The current study showed that the values of *T*_opt_ estimated by SSI model for overall immature stages of *G. pyloalis* was at 24.63 °C, although the highest developmental rate was estimated at 30.7 °C. Based on thermodynamic concepts of SSI model, at *T*_opt_ of 24.63 °C determined for overall immature stages, the maximal active state enzymes involved in the developmental process (Shi et al. 2011b, 2012; Padmavathi et al. 2013). The intrinsic optimum temp at which no enzyme inactivation is hypothesized could represent the most important thermal parameter that determine the fitness of an optimum life history strategy for insects. Therefore, it could be proposed to evaluate the life table parameters of *G. pyloalis* at different temperatures because of lack of such information. In that case, the seasonal prediction of the occurrence as well as severity of the pest would be accurately clarified.

Accordingly, the importance of the seasonal occurrence prediction of the pest for developing management strategies has led to different linear and nonlinear models that describe the developmental rate of *G. pyloalis* in relation to temperature. The results of the present study could provide essential information on temperature-dependent development of *G. pyloalis* and its critical temperatures. Using those valuable information with other ecological data such as intrinsic rate of increase, survival rate and climate factors would enable researchers to predict the population dynamics of *G. pyloalis* for applied IPM implementation.

## References

[iex001-B1] Analytis S. 1981 Relationship between temperature and development times in phytopathogenic fungus and in plant pests: a mathematical model. Agric. Res. (Athens). 5: 133–159.

[iex001-B2] Borgohain A. Battacharjee J. Dutta L. C. Bhattacharya B. Singha T. A. 2015 Influence of climatic factors on infestation and damage of mulberry plant by *Glyphodes pyloalis* Walker in Jorhat (Assam). J. Exp. Zool. India. 18: 821–824.

[iex001-B3] Braman S. K. Sloderbeck P. E. Yeargan K. V. 1984 Effects of temperature on the development and survival of *Nabis americoferus* and *N. roseipennis* (Hemiptera: Nabidae). Ann. Entomol. Soc. Am. 77: 592–596.

[iex001-B4] Briere J. F. Pracros P. Le Roux A. Y. Pierre S. 1999 A novel rate model of temperature dependent development for arthropods. Environ. Entomol. 28: 22–29.

[iex001-B5] Campbell A. Frazer B. D. Gilbert N. Gutierrez A. P. Mackauer M. 1974 Temperature requirements of some aphids and their parasites. J. Appl. Ecol. 11: 431–438.

[iex001-B6] Davidson J. 1944 On the relationship between temperature and the rate of development of insects at constant temperatures. J. Animal. Ecol. 13: 26–38.

[iex001-B7] Honěk A. 1996 Geographical variation in thermal requirements for insect development. Eur. J. Entomol. 93: 303–312.

[iex001-B8] Ikemoto T. 2005 Intrinsic optimum temperature for development of insects and mites. Environ. Entomol. 34: 1377–1387.

[iex001-B9] Ikemoto T. 2008 Tropical malaria does not mean hot environments. J. Med. Entomol. 45: 963–969. 19058618 10.1603/0022-2585(2008)45[963:tmdnmh]2.0.co;2

[iex001-B10] Ikemoto T. Takai K. 2000 A new linearized formula for the law of total effective temperature and the evaluation of line-fitting methods with both variables subject to error. Environ. Entomol. 29: 671–682.

[iex001-B11] Jafari S. H. Fathipour Y. Faraji F. 2012 Temperature-dependent development of *Neoseiulus barkeri* (Acari: Phytoseiidae) on *Tetranychus urticae* (Acari: Tetranychidae) at seven constant temperatures. Insect Sci. 19: 220–228.

[iex001-B12] Karimi-Malati A. Fathipour Y. Talebi A. A. 2014 Development response of *Spodoptera exigua* to eight constant temperatures: Linear and nonlinear modeling. J. Asia Pac. Entomol. 17: 349–354.

[iex001-B13] Khosravi R. Jalali Sendi J. 2010 Biology and demography of *Glyphodes pyloalis* Walker (Lepidoptera: Pyralidae) on mulberry. J. Asia Pac. Entomol. 13: 273–276.

[iex001-B14] Kontodimas D. C. Eliopoulos P. A. Stathas G. J. Economou L. P. 2004 Comparative temperature-dependent development of *Nephus includens* (Kirsch) and *Nephus bisignatus* (Boheman) (Coleoptera: Coccinellidae), preying on *Planococcus citri* (Risso) (Homoptera: Pseudococcidae): evaluation of a linear and various non-linear models using specific criteria. Environ. Entomol. 33: 1–11.

[iex001-B15] Kumar V. Kumar V. Rajadurai S. Babu A. M. Katiyar R. L. Kariappa B. K. Thiagarajan V. Jayaswal K. P. 2002 The chronic architecture and shell structure of *Diaphania pulverulentalis* (Hampson) (Lepidoptera: Pyralidae). Russ. Entomol. J. 11: 307–310.

[iex001-B16] Lactin D. J. Holliday N. J. Johnson D. L. Craigen R. 1995 Improved rate of temperature dependent development by arthropods. Environ. Entomol. 24: 68–75.

[iex001-B17] Madyarov S. R. Khamraev A. S. Otarbaev D. O. Kamita S. G. Hammock B. D. 2006 Comparative effects of wild and recombinant baculoviral insecticides on mp *Glyphodes pyloalis* and mulberry silkworm *Bombyx mori*, pp. 230–231. *In* International Workshop on Silk Handcrafts Cottage Industries and Silk Enterprises Development in Africa, Europe, Central Asia and the Near East, & Second Executive Meeting of Black, Caspian seas and Central Asia Silk Association (BACSA), 6–10 March, Bursa, Turkey.

[iex001-B18] Mathur R. N. 1980 Biology of the mulberry defoliator *Glyphodes pyloalis* (Lepidoptera: Pyralidae). Ind. Forest. Bull. 273: 1–6.

[iex001-B19] Oftadeh M. Jalali Sendi J. Zibaee A. Valizadeh B. 2014 Effect of four varieties of mulberry on biochemistry and nutritional physiology of mulberry pyralid, *Glyphodes pyloalis* Walker (Lepidoptera: Pyralidae). J. Entomol. Acarol. Res. 46: 42–49.

[iex001-B20] Oftadeh M. Jalali Sendi J. Khosravi R. 2015 Life table parameters of *Glyphodes pyloalis* Walker (Lep.: Pyralidae) on four varieties of mulberry *Morus alba* L. (Moraceae). J. Asia Pac. Entomol. 18: 315–320.

[iex001-B21] Padmavathi C. Katti G. Sailaja V. Padmakumari A. P. Jhansilakshmi V. Prabhakar M. Prasad Y. G. 2013 Temperature thresholds and thermal requirements for the development of the rice leaf folder, *Cnaphalocrocis medinalis*. J. Insect Sci. 13: 1–14. 24205891 10.1673/031.013.9601 PMC3835038

[iex001-B22] Ramegowda G. K. Illahi L. Mittal V. Akhter I. Dhar A. Khan M. A. 2012 Influence of weather on the incidence and severity of lesser mulberry pyralid and mulberry looper in Kashmir. Indian J. Entomol. 9: 422–428.

[iex001-B23] Ranjbar-Aghdam H. Fathipour Y. Radjabi G. Rezapanah M. 2009 Temperature dependent development and temperature thresholds of codling moth (Lepidoptera: Tortricidae) in Iran. Environ. Entomol. 38: 885–895. 19508800 10.1603/022.038.0343

[iex001-B24] Roy M. Brodeur J. Cloutier C. 2002 Relationship between temperature and developmental rate of *Stethorus punctillum* (Coleoptera: Coccinellidae) and its prey *Tetranychus mcdanieli* (Acarina: Tetranychidae). Environ. Entomol. 31: 177–187.

[iex001-B25] SAS Institute. 2007 JMP Statistics and Graphics Guide, Release 7. SAS Institute, Cary, NC.

[iex001-B26] Schoolfield R. M. Sharpe P. J. H. Magnuson C. E. 1981 Non-linear regression of biological temperature-dependent rate models based on absolute reaction-rate theory. J. Theor. Biol. 88: 719–731. 6790878 10.1016/0022-5193(81)90246-0

[iex001-B27] Sharpe P. J. H. DeMichele D. W. 1977 Reaction kinetics of poikilotherm development. J. Theor. Biol. 64: 649–670. 846210 10.1016/0022-5193(77)90265-x

[iex001-B28] Shi P. Ge F. Sun Y. Chen C. 2011a A simple model for describing the effect of temperature on insect developmental rate. J. Asia Pac. Entomol. 14: 15–20.

[iex001-B29] Shi P. Ikemoto T. Egami C. Sun Y. Ge F. 2011b A modified program for estimating the parameters of the SSI model. Environ. Entomol. 40: 462–469.

[iex001-B30] Shi P. Li B. L. Ge F. 2012 Intrinsic optimum temperature of the diamondback moth and its ecological meaning. Environ. Entomol. 41: 714–722. 22732631 10.1603/EN12058

[iex001-B31] Shi P. Sandhu H. S. Ge F. 2013 Could the intrinsic rate of increase represent the fitness in terrestrial ectotherms? J. Therm. Biol. 38: 148–151.

[iex001-B32] Tobin C. P. Nagarkatti S. Sauders M. C. 2003 Phenology of grape berry moth (Lepidoptera: Tortricidae) in cultivated grape at selected geographic locations. Environ. Entomol. 32: 340–346.

[iex001-B33] Watanabe H. Kurihara Y. Wang Y. X. Shimizu T. 1988 Mulberry pyralid, *Glyphodes pyloalis*: Habitual host of nonoccluded viruses pathogenic to the silkworm, *Bombyx mori*. J. Invertebr. Pathol. 52: 401–408.

[iex001-B34] Wagner T. L. Wu H. Sharpe P. J. H. Schoolfield R. M. Coulson R. N. 1984 Modeling insect development rate: a literature review and application of a biophysical model. Ann. Entomol. Soc. Am. 77: 208–225.

[iex001-B35] Yazdani E. Jalali Sendi J. Hajizadeh J. 2014 Effect of *Thymus vulgaris* L. and *Origanum vulgare* L. essential oils on toxicity, food consumption, and biochemical properties of lesser mulberry pyralid *Glyphodes pyloalis* Walker (Lepidoptera: Pyralidae). J. Plant Prot. Res. 54: 53–61.

[iex001-B36] Zahiri B. Fathipour Y. Khanjani M. Moharramipour S. Zaluki M. 2010 Preimaginal development response to constant temperatures in *Hypera postica* (Coleoptera: Curculionidae): picking the best model. Environ. Entomol. 39: 177–189. 20146855 10.1603/EN08239

