# Corrigendum

**DOI:** 10.1093/jisesa/iex038

**Published:** 2017-05-10

In the article “Modeling Temperature-Dependent Development of *Glyphodes pyloalis* (Lepidoptera: Pyralidae),” Table 1).

**Table 1.** Developmental time (means ± SE) and survival of *Glyphodes pyloalis* immature stages at constant temperatures

	Temperature (°C)					
Stage	16	20	24	28	30	32
Egg	9.75 ± 0.07a	6.52 ± 0.04b	4.76 ± 0.04c	3.71 ± 0.05d	3.00 ± 0.00e	3.00 ± 0.00e
no (s)	150 (65.71)	112 (70.38)	107 (83.53)	80 (87.50)	106 (77.36)	84 (61.90)
Larva I	8.92 ± 0.18a	4.97 ± 0.09b	3.15 ± 0.07c	2.38 ± 0.10d	2.00 ± 0.00e	2.45 ± 0.10d
no (s)	97 (91.75)	78 (85.90)	89 (100)	59 (100)	82 (100)	52 (100)
Larva II	6.48 ± 0.14a	3.68 ± 0.10b	2.41 ± 0.06c	2.00 ± 0.04d	1.04 ± 0.03e	1.84 ± 0.10d
no (s)	89 (94.38)	67 (98.51)	89 (97.75)	59 (94.92)	82 (100)	52 (100)
Larva III	6.18 ± 0.13a	4.00 ± 0.12b	2.15 ± 0.05c	2.06 ± 0.03cd	1.76 ± 0.06d	1.95 ± 0.10cd
no (s)	84 (90.48)	66 (93.94)	87 (97.70)	56 (100)	82 (95.12)	52 (98.08)
Larva IV	6.72 ± 0.17a	5.53 ± 0.15b	2.64 ± 0.07c	2.44 ± 0.07cd	1.88 ± 0.05e	2.00 ± 0.08de
no (s)	76 (85.53)	62 (96.77)	85 (98.82)	56 (94.64)	78 (94.87)	51 (100)
Larva V	8.22 ± 0.16a	6.18 ± 0.13b	3.72 ± 0.08c	2.87 ± 0.09d	3. 07 ± 0.07d	2.74 ± 0.16d
no (s)	65 (83.08)	60 (100)	84 (96.43)	53 (98.11)	74 (93.24)	51 (86.27)
Larva VI^a^	8.33 ± 1.33a	–	–	–	4.50 ± 1.15b	2.67 ± 0.19b
no (s)	54 (92.59)	–	–	–	69 (98.55)	44 (86.36)
Larvae	37.02 ± 0.34a	24.37 ± 0.31b	14.06 ± 0.12c	11.75 ± 0.13d	10.16 ±0.18e	11.89 ± 0.28d
no (s)	97 (51.55)	78 (76.92)	89 (91.01)	59 (88.14)	82 (82.93)	52 (73.08)
Pre pupa^b^	101.68 ± 11.03	4.64 ± 0.11a	2.28 ± 0.05b	1.94 ± 0.03c	2.11 ± 0.06bc	2.25 ± 0.19bc
no (s)	50 (38)	60 (83.33)	81 (96.30)	52 (100)	68 (92.65)	38 (84.21)
Pupa^b^	24.67 ± 1.61	12.32 ± 0.14a	8.62 ± 0.07b	7.08 ± 0.05c	6.80 ± 0.09c	6.14 ± 0.08d
no (s)	19 (31.57)	50 (94)	78 (87.18)	52 (98.08)	63 (85.71)	32 (65.63)
Immature^b^	134.33 ± 25.19	46.62 ± 0.23a	29.34 ± 0.17b	24.47 ± 0.17c	22.04 ± 0.20d	22.43 ± 0.25d

No, sample size; s, survival (%). Means within rows followed by the same letters are not signiﬁcantly different (*P* < 0.05).

a At 20, 24, and 28 °C larvae completed development in five stadia.

b Comparing the prepupal, pupal, and total developmental times was done without considering of the temperature at 16 °C.

included an error in 6th row; no (s) of larva II. The correct table is below.

The original article has now been corrected.
